# Plasma 25-Hydroxyvitamin D Is Related to Protein Signaling Involved in Glucose Homeostasis in a Tissue-Specific Manner

**DOI:** 10.3390/nu8100631

**Published:** 2016-10-13

**Authors:** Lewan Parker, Itamar Levinger, Aya Mousa, Kirsten Howlett, Barbora de Courten

**Affiliations:** 1 Clinical Exercise Science Research Program, Institute of Sport, Exercise and Active Living (ISEAL), Victoria University, Melbourne VIC 8001, Australia; lewan.parker@live.vu.edu.au (L.P.); Itamar.Levinger@vu.edu.au (I.L.); 2 Monash Centre for Health Research and Implementation, School of Public Health and Preventive Medicine, Monash University, MHRP, 43-51 Kanooka Grove, Clayton VIC 3168, Australia; aya.mousa@monash.edu; 3 Institute for Physical Activity and Nutrition (IPAN), School of Exercise and Nutrition Sciences, Deakin University, Waurn Ponds VIC 3217, Australia; kirsten.howlett@deakin.edu.au; 4 Diabetes and Vascular Medicine Unit, Monash Health, Locked Bag 29, Clayton VIC 3168, Australia

**Keywords:** diabetes, glucose homeostasis, insulin resistance, insulin signaling, vitamin D, 25-hydroxyvitamin D

## Abstract

Vitamin D has been suggested to play a role in glucose metabolism. However, previous findings are contradictory and mechanistic pathways remain unclear. We examined the relationship between plasma 25-hydroxyvitamin D (25(OH)D), insulin sensitivity, and insulin signaling in skeletal muscle and adipose tissue. Seventeen healthy adults (Body mass index: 26 ± 4; Age: 30 ± 12 years) underwent a hyperinsulinemic-euglycemic clamp, and resting skeletal muscle and adipose tissue biopsies. In this cohort, the plasma 25(OH)D concentration was not associated with insulin sensitivity (*r* = 0.19, *p* = 0.56). However, higher plasma 25(OH)D concentrations correlated with lower phosphorylation of glycogen synthase kinase-3 (GSK-3) α^Ser21^ and β^Ser9^ in skeletal muscle (*r* = −0.66, *p* = 0.015 and *r* = −0.53, *p* = 0.06, respectively) and higher GSK-3 α^Ser21^ and β^Ser9^ phosphorylation in adipose tissue (*r* = 0.82, *p* < 0.01 and *r* = 0.62, *p* = 0.042, respectively). Furthermore, higher plasma 25(OH)D concentrations were associated with greater phosphorylation of both protein kinase-B (Akt^Ser473^) (*r* = 0.78, *p* < 0.001) and insulin receptor substrate-1 (IRS-1^Ser312^) (*r* = 0.71, *p* = 0.01) in adipose tissue. No associations were found between plasma 25(OH)D concentration and IRS-1^Tyr612^ phosphorylation in skeletal muscle and adipose tissue. The divergent findings between muscle and adipose tissue with regard to the association between 25(OH)D and insulin signaling proteins may suggest a tissue-specific interaction with varying effects on glucose homeostasis. Further research is required to elucidate the physiological relevance of 25(OH)D in each tissue.

## 1. Introduction

Vitamin D, in its hydroxylated form, 25-hydroxyvitamin D (25(OH)D), and its biologically active form, 1,25-dihydroxyvitamin D (1,25(OH)_2_D), is an important regulator of calcium, phosphorus, and bone metabolism [1]. Vitamin D is primarily produced in the skin in response to ultraviolet B radiation but can also be obtained from diet (e.g., high-fat fish, liver, or fortified milk) or supplementation in the form of vitamin D_2_ or D_3_ [2,3]. Vitamin D is transported to the liver in blood via vitamin D binding proteins where it is hydroxylated to form 25(OH)D, the most abundant circulating form of vitamin D. 25(OH)D is then transported to the kidney and hydroxylated to the hormonally active form of 1,25(OH)_2_D which is suggested to elicit many, if not all, of the biological actions of vitamin D [4]. Vitamin D status is primarily measured via serum/plasma 25(OH)D due to the substantially greater half-life and abundance in serum/plasma compared to 1,25(OH)_2_D and the absence of confounding factors such as elevated 1,25(OH)_2_D concentrations in vitamin D-insufficient and -deficient populations [5]. Although variations permeate the literature, vitamin D status is generally considered to be deficient when 25(OH)D serum concentrations are <25 nmol/L, insufficient when between 25 and 49 nmol/L, and sufficient when ≥50 nmol/L [6]. Vitamin D deficiency is linked to the development of several pathologies including cancer, autoimmune diseases, infectious diseases, and inflammatory conditions [7]. In addition, previous reports suggest that vitamin D plays a role in glycemic control [8,9] which can be improved through vitamin D supplementation [8,9,10]; however, findings are contradictory [11,12,13]. Potential cellular pathways are unclear, but may occur through direct binding of vitamin D to vitamin D receptors and activation of downstream signaling proteins; through increased gene expression of the insulin receptor; and indirectly through calcium regulation and subsequent downstream effects on glucose homeostasis signaling proteins [14,15,16,17]. Previous reports indicate that vitamin D supplementation in high-fat diet–fed mice attenuates weight gain and increases transcriptional activity of the insulin receptor substrate-1 (IRS-1) in skeletal muscle but not adipose tissue [18]. Likewise, 1,25(OH)_2_D treatment in C2C12 cells rescues diet-induced insulin resistance via decreased IRS-1 serine phosphorylation and increased IRS-1 tyrosine and protein kinase-B (Akt) serine phosphorylation [19]. Further, 1,25(OH)_2_D also participates in skeletal muscle cell proliferation and differentiation through a phosphoinositide 3-kinase/Akt-dependent signaling pathway [20] that is known to regulate glycemic control [21]. However, to the best of our knowledge, there is no human data on insulin signaling. Therefore, the aim of this study was to test the hypothesis that vitamin D status and 25(OH)D in humans is related to insulin signaling proteins in both skeletal muscle and adipose tissue.

## 2. Materials and Methods

This study is a subset of a larger study of 111 healthy normoglycemic adults, where we reported a positive association between plasma 25(OH)D concentration and insulin sensitivity measured by hyperinsulinemic-euglycemic clamp [22]. Due to the invasive nature of skeletal muscle and subcutaneous adipose tissue sampling in humans, the sample size is limited to seventeen adults (ten males and seven females) who had participated in the larger study. Detailed methodology for medical pre-screening, insulin sensitivity and protein analysis have previously been reported [22]. In brief, biopsies of subcutaneous adipose and vastus lateralis muscle were performed at rest prior to the hyperinsulinemic-euglycemic clamp after a 12 h overnight fast, under local anesthesia, and using standard aseptic techniques. Following a 7 mm skin incision to cut the fascia prior to the muscle biopsy, a side-cutting muscle biopsy needle was passed through to obtain ~100 mg of muscle tissue. Using a 50 mL plastic syringe attached to a 13 gauge aspiration needle, adipose tissue was obtained from the abdominal area, 1–2 cm superior to McBurney’s point. Both muscle and adipose tissues were immediately placed in liquid nitrogen and then stored at 80 °C.

Insulin sensitivity was measured via a 2 h hyperinsulinemic-euglycemic clamp. Insulin was infused at a constant rate of 40 mU/m_2_/min and the glucose infusion rate (GIR) was averaged over the last 40 min of the insulin clamp and expressed relative to serum insulin levels (m-value; mg/kg/min). Fasting blood samples for 25(OH)D analysis were drawn prior to the start of the insulin clamp. Primary antibodies specific for phosphorylation of glycogen synthase kinase (GSK-3) α/β^Ser21/9^ (Cell Signaling Technology, Danvers, MA, USA), protein kinase-B (Akt^Ser473^; Cell Signaling Technology, Danvers, MA, USA), serine phosphorylation of human insulin receptor substrate 1 (IRS-1^Ser312^; Biosource), and tyrosine phosphorylation of IRS-1^Tyr612^ (Biosource), were used to quantify muscle and adipose tissue protein as per methods previously reported [23].

Vitamin D assays were performed using a DiaSorin Liaison Analyser (DiaSorin Inc., Stillwater, MN, USA), which uses the direct competitive chemiluminescent immunoassay method (CLIA) to quantitatively determine the total 25(OH)D in plasma. During the first incubation, 25(OH)D is dissociated from its binding proteins and binds to the specific antibody on the solid phase. After 10 min, the tracer (vitamin D linked to an isoluminol derivative) is added. Following a second incubation, the unbound material is removed with a wash cycle and the starter reagent is added to initiate a flash chemiluminescent reaction. The light signal emitted is measured by a photomultiplier as relative light units (RLU) and is inversely proportional to the concentration of 25(OH)D present in the sample. Inter- and intra-assay coefficients of variation (CV) using the Diasorin method were <10% and <4%, respectively.

All data were log transformed to approximate normal distribution prior to analysis. Unpaired *t*-tests assuming equal variance were conducted to compare mean insulin sensitivity values between groups of different vitamin D status. Correlations of combined data, and data stratified by deficient/insufficient (25(OH)D < 50 nmol/L) and sufficient (25(OH)D ≥ 50 nmol/L) vitamin D status, were analyzed using linear regression analysis. Due to difficulty in adipose tissue and skeletal muscle sampling in humans, samples were not available for some protein analysis for all subjects (*n* = 11–16). Cook’s Distance was used as a measure of influence where observations greater than 4/N Cook’s D were excluded from correlation analysis [24]. All data are reported as mean ± SD and all statistical analyses were conducted at the 95% level of significance (*p* < 0.05).

Ethical approval was obtained from the Alfred Hospital Ethics Committee and the Monash University Human Research Ethics Committee and complied with the Declaration of Helsinki (2013) [25] All participants provided written informed consent prior to participation. (The ethical approval code is HREC 131/04).

## 3. Results

Participant characteristics are reported in Table 1.

### 3.1. Plasma 25(OH)D and Insulin Sensitivity

**Figure 1:**
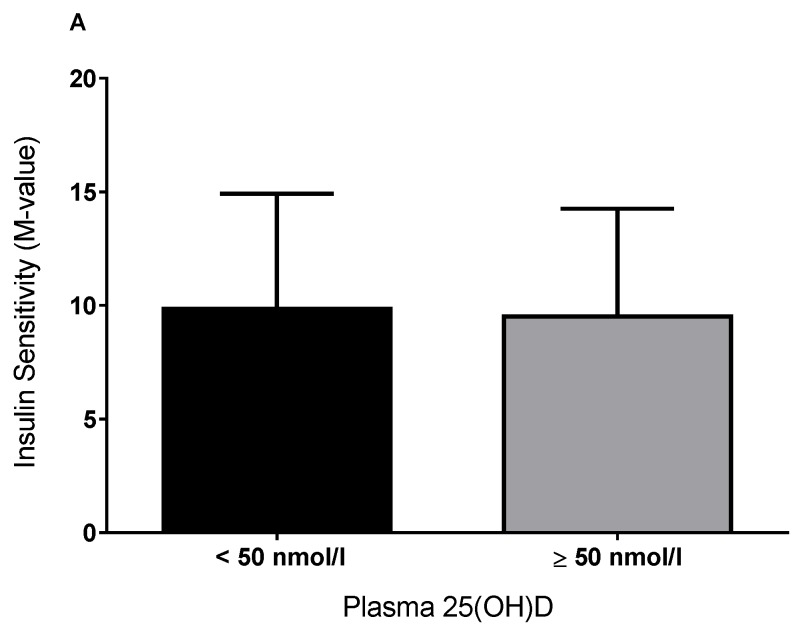
Insulin sensitivity (M-value; A), as measured by hyperinsulinemic-euglycemic clamp, stratified by deficient/insufficient and replete vitamin D status.

As previously reported, in the larger sample of 111 individuals (66 males, 45 females, age = 31.1 ± 9.2 years, body mass index (BMI) = 30.2 ± 5.1 kg/m^2^), there was a positive association between 25(OH)D and insulin sensitivity (*r* = 0.20, *p* = 0.04). In the subgroup of 17 individuals where skeletal muscle and adipose tissue was available, the M-value was not significantly different between vitamin D–deficient/–insufficient (30 ± 10 nmol/L) and vitamin D-sufficient (82 ± 28 nmol/L) individuals (*p* = 0.9; Figure 1). The plasma 25(OH)D concentration was not associated with insulin sensitivity (M-value) (*r* = 0.10, *p* = 0.69), even after adjustment for age, BMI, fat mass, sex, and vitamin D status (*r* = 0.19, *p* = 0.56).

### 3.2. Plasma 25(OH)D and Protein Regulators of Insulin Signaling

**Figure 2:**
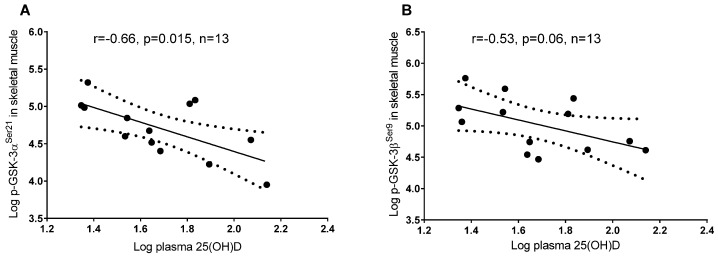

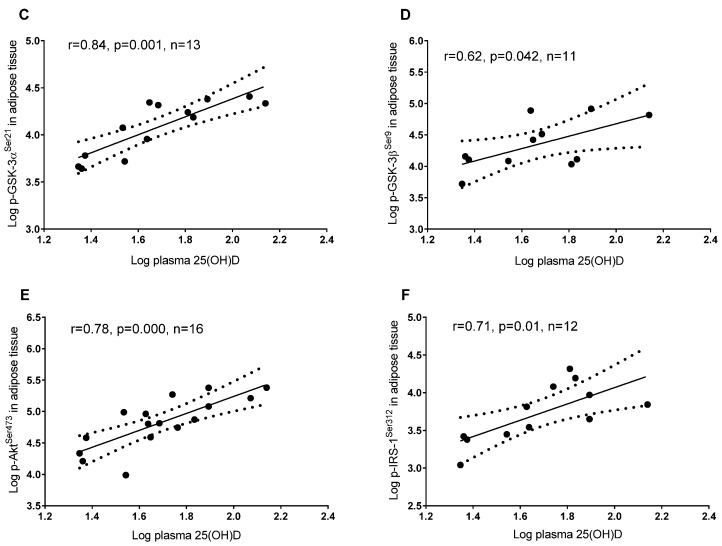
Linear regression between plasma vitamin D (25(OH)D) and insulin signaling proteins in adipose tissue and skeletal muscle: (**A**) Plasma 25(OH)D and phosphorylation of GSK-3 α^Ser21^ in skeletal muscle; (**B**) Plasma 25(OH)D and phosphorylation of GSK-3 β^Ser9^ in skeletal muscle; (**C**) Plasma 25(OH)D and phosphorylation of GSK-3 α^Ser21^ in adipose tissue; (**D**) Plasma 25(OH)D and phosphorylation of GSK-3 β^Ser9^ in adipose tissue; (**E**) Plasma 25(OH)D and phosphorylation of Akt^Ser473^ in adipose tissue; (**F**) Plasma 25(OH)D and phosphorylation of IRS-1^Ser312^ in adipose tissue.

**Figure 3:**
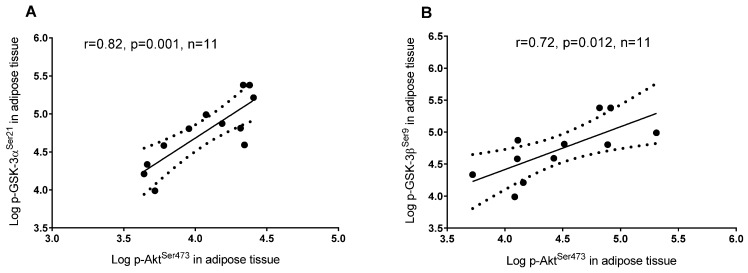
Linear regression between: (**A**) Phosphorylated Akt^Ser473^ and phosphorylation of GSK-3 α^Ser21^ in adipose tissue; (**B**) Phosphorylated Akt^Ser473^ and GSK-3 β^Ser9^ in adipose tissue.

In skeletal muscle, a higher plasma 25(OH)D concentration was associated with lower phosphorylation of GSK-3 α^Ser21^ and β^Ser9^, but the latter did not reach significance (*p* = 0.06), whereas in adipose tissue 25(OH)D was associated with a higher phosphorylation of GSK-3 α^Ser21^ and β^Ser9^ (Figure 2). In adipose tissue, a higher plasma 25(OH)D concentration was associated with greater phosphorylation of Akt^Ser473^ and IRS-1^Ser312^ (Figure 2), whereas these associations were not detected in skeletal muscle (*p* = 0.99 and *p* = 0.56, respectively; data not shown). Tyrosine phosphorylation of IRS-1^Tyr612^ in adipose tissue and skeletal muscle did not correlate with 25(OH)D concentrations (*p* = 0.95 and *p* = 0.3, respectively; data not shown). GSK-3 α^Ser21^ and β^Ser9^ phosphorylation was associated with higher phosphorylated Akt^Ser473^ in adipose tissue (Figure 3) but not in skeletal muscle (*p* = 0.93 and *p* = 0.6, respectively; data not shown).

**Figure 4:**
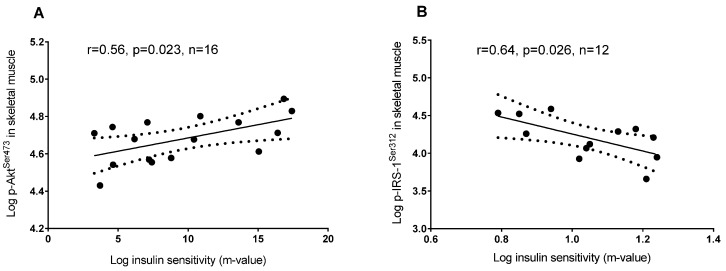
Linear regression between: (**A**) Insulin sensitivity (M-value) and Akt^Ser473^ phosphorylation in skeletal muscle; (**B**) Insulin sensitivity (M-value) and IRS-1^Ser312^ phosphorylation in skeletal muscle.

Insulin sensitivity was not associated with phosphorylation of GSK-3 α^Ser21^ and β^Ser9^ in skeletal muscle (*p* = 0.83 and *p* = 0.92, respectively; data not shown) or adipose tissue (*p* = 0.28 and *p* = 0.42, respectively; data not shown). On the other hand, insulin sensitivity was associated with higher phosphorylation of Akt^Ser473^ and lower phosphorylation of IRS-1^Ser312^ in skeletal muscle (Figure 4), but not in adipose tissue (*p* = 0.96 and *p* = 0.23, respectively; data not shown).

## 4. Discussion

We report that the association between 25(OH)D and insulin signaling proteins appears to be tissue specific. Differential associations were detected between 25(OH)D concentration and key glucose homeostasis signaling proteins IRS-1^Ser312^, GSK-3 α/β^Ser21/9^, and Akt^Ser473^, in skeletal muscle and adipose tissue.

Considering the well-known role of calcium in glycemic control [16] and given that vitamin D is a calcitropic hormone, it was hypothesized that vitamin D may also play a role in glucose metabolism [8,9,10]. We previously reported that plasma 25(OH)D was related to insulin sensitivity in a larger study [22]; however, this relationship was not present in the subgroup analysis of participants who underwent skeletal muscle and adipose tissue sampling. Similar to our findings, previous studies using the hyperinsulinemic-euglycemic clamp with similar sample sizes have reported that vitamin D supplementation in both vitamin D-deficient/-insufficient [13] and replete individuals [26] has a modest effect on insulin sensitivity. In contrast, vitamin D supplementation in insulin-resistant women has been reported to reduce insulin resistance when endpoint 25(OH)D concentrations were equal to or exceeded 80 nmol/L [27], suggesting that a potentially higher physiological limit of 25(OH)D is required to influence glycemic control. In the current study, no supplementation was used, only one participant exhibited a plasma 25(OH)D concentration above 80 nmol/L, and a small sample size may have contributed towards the lack of significant findings which have previously been reported in epidemiological studies [9,10]. Nevertheless, our findings are in accordance with intervention studies which have reported that improving vitamin D status plays a limited role in glucose metabolism [11,13,26,28,29,30,31,32,33].

An important finding of the current study was the strong correlation between the plasma 25(OH)D concentration and key insulin signaling proteins involved in glucose homeostasis, which appear to occur in a tissue-specific manner. Previous reports suggest that vitamin D deficiency may influence glucose homeostasis through increased intracellular calcium leading to aberrant insulin signaling and attenuation of downstream insulin signaling enzymes such as glycogen synthase and the glucose transporter 4 (GLUT 4) [8,15,17,34,35]. We report the novel finding that higher plasma 25(OH)D concentrations were associated with greater serine phosphorylation of GSK-3 α/β and its upstream regulator Akt in adipose tissue, and lower serine phosphorylation of GSK-3 α/β in skeletal muscle. GSK-3 is highly active under resting conditions in the absence of insulin and inhibits glycogen synthase activity, glycogen synthesis and subsequent glucose uptake, via serine phosphorylation on residues 21 and 9 [36,37]. The association of higher plasma 25(OH)D with lower GSK-3 serine phosphorylation in skeletal muscle suggests greater basal inhibition of glycogen synthase activity, glycogen synthesis, and glucose uptake, whereas higher GSK-3 serine phosphorylation in adipose tissue suggests upregulation of this pathway. This inverse relationship between 25(OH)D and serine phosphorylated GSK-3 α/β in muscle and adipose tissue may indicate a tissue-specific role for 25(OH)D in GSK-3 function. Previous reports indicate that higher protein expression of GSK-3 (often elevated in patients with type 2 diabetes) is related to lower insulin action in skeletal muscle, whereas GSK-3 in adipose tissue is less likely to play a role in whole body glucose disposal [38]. It is also possible that 25(OH)D may be involved in other GSK-3–mediated cellular processes independent of glucose regulation, such as gene transcription, mRNA translation, cytoskeletal regulation, cell cycle progression, and apoptosis [39]. Further research is required to investigate the potential role of vitamin D status and GSK-3 function in human skeletal muscle and adipose tissue.

Increased phosphorylation of IRS-1^Ser312^ is implicated with impaired insulin signaling and glucose uptake in part through IRS-1 degradation, impaired IRS-1 tyrosine phosphorylation, and attenuation of insulin-stimulated glucose uptake [40]. We report that a higher plasma 25(OH)D concentration was associated with greater phosphorylation of IRS-1^Ser312^ in adipose tissue but not in skeletal muscle tissue. In addition, 25(OH)D concentration was not associated with basal tyrosine phosphorylation of IRS-1^Tyr612^, a positive regulator of insulin-stimulated glucose uptake [41], in either tissue. Our findings contradict previous reports which have reported a role for 1,25(OH)_2_D in the enhancement of insulin signaling and insulin sensitivity in non-human studies [18,19], although the measurement of 25(OH)D and basal insulin signaling in the current study limits comparisons. Prada, Zecchin [42] reported that IRS-1^Ser312^ phosphorylation in skeletal muscle likely plays a greater role in insulin sensitivity than in adipose tissue. Indeed, we report that greater phosphorylation of IRS-1^Ser312^ in skeletal muscle only was related to lower whole body insulin sensitivity. Thus, in the context of the current findings, the plasma 25(OH)D concentration does not appear to be related to basal IRS-1^Tyr612^ phosphorylation or IRS-1^Ser312^ phosphorylation in skeletal muscle; however, future research is required to elucidate the physiological relevance of 25(OH)D and IRS-1^Ser312^ signaling in adipose tissue.

This study has potential limitations which include a relatively small sample size, and the measurement of insulin signaling proteins under basal fasted conditions and not during or after insulin stimulation. The inclusion of populations who are insulin resistant, have type 2 diabetes, and/or exhibit a greater range of vitamin D status would allow for greater exploration of the relationship between vitamin D status, and insulin sensitivity and protein signaling. Future studies are required to confirm these findings with larger and more diverse populations. Interventional studies in vitamin D–deficient and insulin-resistant individuals would allow for greater exploration of the physiological role and relevance of 25(OH)D and insulin signaling proteins. It is possible that vitamin D status is subsidiary to insulin resistance and an expression of ill health rather than a primary moderator of glycemic control [43]. However, current evidence is equivocal and further high-quality randomized controlled trials are required [9].

## 5. Conclusions

In conclusion, plasma 25(OH)D was strongly associated with phosphorylation of insulin signaling proteins in a tissue-specific manner. The clinical implications of these findings need to be examined with adequately powered randomized controlled trials.

## Figures and Tables

**Table 1:** Participant characteristics.

Variable	All	Males	Females
Age (years)	30 ± 12 (18–49)	28 ± 10 (18–46)	33 ± 13 (21–49)
Height (cm)	172 ± 6 (173–199)	175 ± 5 (167–185)	167 ± 6 (159–176)
Weight (kg)	78 ± 11 (53–99)	81 ± 10 (66–99)	72 ± 12 (53–91)
BMI (kg∙m^−2^)	26 ± 4 (21–34)	27 ± 4 (22–34)	26 ± 4 (21–31)
Plasma 25(OH)D (nmol/L)	57 ± 31 (22–138)	65 ± 35 (23–138)	47 ± 20 (22–78)
Vitamin D status (25(OH)D)			
Deficient: <25 nmol/L	3	1	2
Insufficient: 25–49 nmol/L	6	4	2
Sufficient: ≥50 nmol/L	8	5	3
GIR per unit of insulin (M-value; mg/kg/min)	10 ± 5 (3–17)	9 ± 3 (4–15)	10 ± 6 (3–17)

Data presented as mean ± SD (Range); BMI, body mass index; 25(OH)D, 25-hydroxyvitamin D, GIR, glucose infusion rate.
